# Analysing the expenditure on childbearing: a community-based cross-sectional study in rural areas of Punjab (India)

**DOI:** 10.1186/s12913-021-06075-2

**Published:** 2021-01-21

**Authors:** Niharika Mahajan, Baljit Kaur

**Affiliations:** 1 Research Scholar, Punjab School of Economics, Guru Nanak Dev University, Amritsar, India; 2 Assistant Professor, Punjab School of Economics, Guru Nanak Dev University, Amritsar, India

**Keywords:** Maternity expenditure, OOPE, Catastrophic, JSSK

## Abstract

**Background:**

A vast array of literature has established that high maternity expenditure precludes women from accessing health services. Further, this maternity expenditure takes catastrophic form, forcing individuals or households to significantly lower their standard of living now or at some time in future. The present study analyses expenditure on childbearing in rural areas of one of the richest and top performer states on health parameters in India, namely Punjab along with examining the determinants of catastrophic expenditure. It also attempts to examine the implementation of Janani Shishu Suraksha Karyakaram (JSSK) which entitles pregnant women to free maternity services in public health facilities.

**Methods:**

A cross-sectional study was conducted in rural areas of Punjab involving 420 recently delivered women, who were questioned about their socio-economic attributes and expenditure incurred in the process of childbearing using face to face, semi-structured interviews. Employing logistic regression, an attempt has been made to understand the determinants of catastrophic maternity expenditure, i.e., expenditure exceeding 10% of annual household income.

**Results:**

Of the 420 respondents surveyed, 96.7% reported bearing expenditure on childbearing, irrespective of the type of health facility used and 25% respondents spent catastrophically. On an average, respondents have spent US$62.87 on antenatal care, US$112.86 on delivery and US$6.55 on postnatal care. The results of multivariable analysis reveal that respondents belonging to general category (non reserve category), lower wealth quintiles and using private health facilities have higher odds of incurring catastrophic expenditure. At the same time, poor quality of care at government hospitals and inability of public health staff to provide timely treatment are the driving forces for utilizing private health facilities. Even in the presence of free maternity scheme at government hospitals, respondents on an average spent US$55.22 on availing maternity services.

**Conclusion:**

The study shows that risk of bearing catastrophic expenditure and being pushed down to abject poverty is higher for respondents who are already at the bottom of wealth quintiles. The policy imperative has to swing towards upgrading the creaky health infrastructure and addressing the issues of poor accountability and corruption at government hospitals, along with thwarting unregulated expansion of private health sector.

**Supplementary Information:**

The online version contains supplementary material available at 10.1186/s12913-021-06075-2.

## Background

Over the years, the meaning of term development has undergone a change with development being no longer measured by rapid gains in overall and per capita GNP alone; it must bring an improvement to the quality of life. A better quality of life calls for better education, higher standards of health and nutrition, less poverty, a cleaner environment, more equality of opportunity, greater individual freedom, and a richer cultural life [1]. Health is playing an increasing role in equitable and sustainable development of countries and its role has been identified in the framing of Millennium Development Goals (2000–2015) and Sustainable Development Goals (2016–2030). Investment in health is an important precondition for breaking the shackles of poverty, especially in developing countries [2]. Poverty and poor health share an intricate relationship where one compounds the other; poverty hinders access to quality food and health services, and the costs (direct and indirect) of seeking health care in case of illness, trap poor people in a downward spiral from which they may never recover. With 800 million people in the world spending at least 10% of their household budgets to receive healthcare, about 100 million people are plunged into extreme poverty [3]. Out-of-pocket payments although inefficient, inequitable and regressive, continue to remain the prime means of financing healthcare in most developing nations. Providing cost-efficient health services to the poor becomes an important countervailing strategy to reduce poverty [4].

When discussing about the need to make healthcare accessible to the people of the world under Universal Health Coverage, the health of women and children deserves special attention, and in particular, the health of mothers [5]. Maternal and child health is one such domain which exercises an influence on the health and quality of life of each generation and more so in developing world. Death and illness resulting from the complications during pregnancy and childbirth are unsettling and disturbing symptoms of poverty and disadvantage. Investing in the health of women and children enables them to secure their fundamental rights along with being a cost-effective approach to rein in poverty and galvanize productivity and growth. WHO in its World Health Report, 2005 [6] identified poor maternal conditions as the fourth leading cause of the deaths of women globally after HIV AIDS, malaria and tuberculosis. A large number of maternal deaths could be avoided if basic maternal care in the form of skilled birth attendant; prevention and treatment of complications during pregnancy, delivery and post-partum period; and post-natal family planning and basic neonatal care is made available. However, high maternity related health care spending proves deleterious for the utilization of quality health care during pregnancy and childbirth and turns catastrophic for many, especially in low-income setting [7–12]. Failure to access health services due to cost barrier pose a considerable challenge for a country like India which is already battling with a glaring share in global maternal deaths. A study conducted post the implementation of JSSK using data from 71st round of National Office argued that high expenditure on maternal health care services plunged 47% women into poverty in India [12]. To minimize the childbearing expenses incurred by women, the Government of India launched a free maternity scheme called Janani Shishu Suraksha Karyakaram (JSSK) in the year 2011 under which free and cashless maternity services in public health facilities and any medical treatment of sick neonate up to 30 days of birth are made available. Entitlements offered under JSSK include zero cost delivery (normal or caesarean); free drugs and consumables; purvey of free diet to the mother (up to 3 days for normal delivery and up to 7 days for caesarean section); provision of free blood; availability of free transport to and back from health facility and between facilities in case of referral; and exemption from all sorts of user charges.

Against this background, the study attempts to achieve the following objectives:

- To examine the type of health facilities used by the respondents for availing maternity services and the nature of out of pocket expenditure on childbearing in the rural areas of Punjab.
- To study the incidence and correlates of catastrophic maternal expenditure.
- To analyse the effect of JSSK on easing out the financial burden on households due to childbirth.

## Methods

Deploying multistage sampling technique, in the first stage, random selection of districts was made till the point where the combined rural female population of the reproductive age (15 to 49 years) in the chosen districts makes at least 50 % of the total rural reproductive female population of Punjab. Accordingly, seven districts out of 22 districts were selected, namely, Amritsar, Gurdaspur, Hoshiarpur, Jalandhar, Ludhiana, Patiala and Tarn Taran. The second stage consisted of randomly choosing two blocks from each district and in the third stage two villages from each block were selected making a total of 28 villages. Community Health Workers in every village maintain a list of all pregnant women and lactating mothers and using these lists in the final stage, 15 respondents from each village who had a live birth in one year preceding the date of survey were randomly selected. All recently delivered women who were approached for the study agreed to participate in the interview process. Face to face interviews using pre-tested semi-structured interview schedule were conducted with these 420 respondents in their home settings by the lead author (NM), a female doctorate research fellow. Interview schedule was developed using guidance from the questionnaire used in National Family Health Surveys in India. Interview schedule developed for the study is presented as Additional File 1. The interviews were conducted during January, 2018 to April, 2018 and each interview lasted for approximately 20–30 min. Respondents were informed about the objectives of the study and their written consent was obtained. The questions were translated into the local language by the interviewer and information on socio-economic attributes and the expenditure incurred at aggregated and disaggregated levels on availing maternity services i.e. total expenditure and expenditure according to the component of care were solicited and recorded on the interview schedule after translating in English. Respondents were usually accompanied by the elderly female of the household at the time of interview.

A wide range of literature demonstrates positive correlation between health and socio-economic status [13] where socio-economic status dissimilarly impacts the care seeking behavior [14]. Using data on asset ownership and housing characteristics, a proxy indicator called wealth index is created to measure socio-economic status as being used by District Household Surveys worldwide. As proposed by Filmer and Pritchett (2001) [15], principal component analysis (PCA) is employed to determine the asset weights for computing the index. In the present study questions related to ownership of house, land, farm animals, kind of dwelling (kutcha, semi-pucca or pucca), number of rooms for sleeping, availability of separate kitchen, fuel used for cooking, access to improved source of drinking water and sanitation facility, treatment of water before drinking, availability of own toilet, ownership of consumer durables (TV, fridge, bike, washing machine, cooler, ac, car, cycle, any other) have been put to the respondents. Using the index derived from PCA, respondents were classified into five wealth quintiles where the first quintile represents the most poor and the fifth quintile represents the least poor.

Health expenditure is termed catastrophic if its proportion in household income or total expenditure crosses some threshold level. In the present study, a household is taken to have incurred catastrophic expenditure if the childbearing necessitates at least 10% of the household’s annual income in expenses [16] and a dichotomous variable is constructed which takes value 1 when expenditure is catastrophic and 0 otherwise.

$$ {I}_c=1\  if\ \frac{h_i}{x_i}\ast 100\ge 10 $$

$$ {I}_c=0\  otherwise $$

where I_c_ is an indicator variable, x_i_ is the total annual income of the household and h_i_ is the out of pocket expenditure incurred on maternity services. Maternity expenditure includes both direct costs of treatment (drugs and medicine supplies, laboratory and diagnostics, consultation fees, room rent, transportation, etc.) and indirect costs of treatment in the form of lost productive labour time and thus labour earnings of mother as well as the caregivers. Household income is the sum of income earned by the members of household from all sources including pension and remittance from abroad. The choice of explanatory variables is based on existing literature [7, 12, 17, 18]. Multivariable logistic regression model is fitted to assess the association between the outcome variable (odds of incurring catastrophic health expenditure) and the selected independent variables (district, religion, social group, proxy of socio-economic status, maternal age, educational qualification of the mother, place of antenatal care, place of delivery and place of post natal care).

## Results

### Pattern of utilization of facilities for maternity services

A detailed distribution of respondents by their socio-economic characteristics and place of availing maternity services is presented in Table 1.

**Table 1:** Distribution of respondents by place of ANC, delivery and PNC

Background Characteristics	Place of ANC			Place of Delivery			Place of PNC		
	Government	Private	Both	Government	Private	Home	None^#^	Government	Private
**Age Groups**									
Less than 20 years	15 (83.3)	1 (5.6)	2 (11.1)	10 (55.6)	6 (33.3)	2 (11.1)	7 (38.9)	8 (44.4)	3 (16.7)
20 to 30	278 (78.5)	26 (7.3)	50 (14.1)	39 (67.5)	104 (29.4)	11 (3.1)	162 (45.8)	108 (30.5)	84 (23.7)
30 and above	29 (60.4)	9 (18.8)	10 (20.8)	22 (45.8)	26 (54.2)	0 (0.0)	16 (33.3)	11 (22.9)	21 (43.8)
**Education**									
Nil	67 (94.4)	1 (1.4)	3 (4.2)	60 (84.5)	7 (9.9)	4 (5.6)	44 (62.0)	23 (32.4)	4 (5.6)
Up to Primary	56 (87.5)	3 (4.7)	5 (7.8)	49 (76.6)	11 (17.2)	4 (6.3)	36 (56.3)	21 (32.8)	7 (10.9)
Up to Secondary	135 (80.4)	11 (6.5)	22 (13.1)	116 (69.0)	48 (28.6)	4 (2.4)	69 (41.1)	58 (34.5)	41 (24.4)
Senior Secondary and above	64 (54.7)	21 (17.9)	32 (27.4)	46 (39.3)	70 (59.8)	1 (0.9)	36 (30.8)	25 (21.4)	56 (47.9)
**Birth Parity**									
Less than 2	123 (74.5)	15 (9.1)	27 (16.4)	101 (61.2)	60 (36.4)	4 (2.4)	56 (33.9)	58 (35.2)	51 (30.9)
2 to 4	173 (76.2)	20 (8.8)	34 (15.0	148 (65.2)	70 (30.8)	9 (4.0)	113 (49.8)	62 (27.3)	52 (22.9)
More than 4	26 (92.9)	1 (3.6)	1 (3.6)	22 (78.6)	6 (21.4)	0 (0.0)	16 (57.1)	7 (25.0)	5 (17.9)
**Wealth Quintiles**									
First	78 (92.9)	2 (2.4)	4 (4.8)	67 (79.8)	10 (11.9)	7 (8.3)	51 (60.7)	28 (33.3)	5 (6.0)
Second	73 (86.9)	2 (2.4)	9 (10.7)	63 (75.0)	20 (23.8)	11 (1.2)	36 (42.9)	34 (40.5)	14 (16.7)
Third	68 (81.2)	3 (3.5)	13 (15.3)	54 (64.7)	28 (32.9)	2 (2.4)	37 (43.5)	24 (28.2)	24 (28.2)
Fourth	60 (71.4)	8 (9.5)	16 (19.0)	54 (64.3)	27 (32.1)	3 (3.6)	34 (40.5)	27 (32.1)	23 (27.4)
Fifth	43 (50.6)	121 (25.3)	20 (24.1)	32 (38.6)	52 (61.4)	–	27 (32.5)	14 (16.9)	43 (50.6)
**Overall**	**(76.6)**	**(8.6)**	**(14.8)**	**(64.5)**	**(31.4)**	**4.0)**	**(45.0)**	**(29.5)**	**(25.5)**

^#^ includes those women who did not have any checkup post discharge from facility and those who delivered at home

Source: Primary Data

Figures in parenthesis are respective percentages

Antenatal checkups are instrumental in the identification and management of obstetric complications during pregnancy. All the respondents in the study have received at least one antenatal checkup with more than 96% respondents having received at least four antenatal checkups and more than 99% respondents having received at least one tetanus toxoid injection. Consumption of 100 and more iron folic tablets or its equivalent syrup was relatively poor with 30.2% consuming less than the required amount. In the present study, government facilities have remained the major source for obtaining antenatal checkups where 76.6% of the respondents solely used these facilities to avail antenatal care. Further over 14% respondents have used both government and private facilities for getting their prenatal checkups while only 8.6% respondents have reported to use private facilities alone. Among all the wealth quintiles, there is a preponderance of government facilities over private facilities for seeking ante natal care.

The proportion of institutional deliveries in the present study is 96%. Even among the health facilities used for delivering children, government hospitals have occupied the dominant position (64.5%) followed by the share of private deliveries (31.4%). Among different age groups, preference for public deliveries is high among age groups of less than 20 years and 20 to 30 years; women in the higher age bracket of 30 and above reported to prefer private deliveries. Similarly, there exists a general proclivity towards private facilities among better educated and wealthier respondents. Of the respondents with senior secondary and above educational qualification, 59.8% have private deliveries. With increase in birth parity, use of government health facilities for antenatal care and delivery increases. However, it is also observed that as we move from lower birth parity to higher, the proportion of women with incomplete postnatal care is also on rise. While the proportion of private deliveries for the third wealth quintile is 26.2%, it increases to 35.6 for fourth quintile and to 43.2% for fifth quintile. The share of caesarean section deliveries among the total government deliveries stands at 29% while the share increases to 59.5% in case of private deliveries. Of the respondents who had institutional deliveries, 98.5% received their first post natal checkup within 24 h of delivery and majorly the place of post natal checkup has been the place of delivery. Post partum period is a crucial period in the life of mothers and newborns since majority of the deaths of mothers and infants occur during the first month post birth. However, the study finds that out of 420 respondents covered in the study, 17 respondents who delivered at home did not avail any post natal checkup. Further, 172 respondents with institutional deliveries did not receive any post natal checkup after discharge from hospital. A higher percentage of such women exist in the age group of 20 to 30 years and amongst women at lower rungs of education ladder. Even the socio-economic status does not have much influence on the up take of post natal services since a significant percentage of women without adequate number of post natal checkups exist amongst all wealth quintiles. Among the remaining 231 respondents, 124 have availed post natal care from public health facilities and 107 respondents have availed post natal care from private health facilities.

### Average (mean) expenditure per childbirth

The mean direct expenditure incurred by respondents on ANC, delivery and PNC according to background characteristics is delineated in Table 2.

**Table 2:** Average Expenditure (Direct cost of treatment) per childbirth (ANC, delivery and PNC); and Catastrophic Expenditure by background characteristics of respondents

Background Characteristics	ANC expenditure (Rs.)	Delivery Expenditure (Rs.)	PNC Expenditure (Rs.)	Total Expenditure (Rs.)	Catastrophic Maternal Expenditure (%)
**Age Groups**					
Less than 20	2297.22 (US $33.29)* (SD 3617.95)	3969.44 (US $57.53) (SD 7254.80)	61.11 (US $0.89) (SD 181.14)	6327.78 (US $91.71) (SD 9892.63)	16.7
20 to 30	4184.16 (US $60.64) (SD 7963.33)	7185.14 (US $104.14) (SD 13587.74)	450.21 (US $6.52) (SD 1165.57)	11,820.01 (US $171.30) (SD 17819.39)	22.6
30 and above	6236.46 (US $90.38) (SD 10684.80)	13,659.90 (US $197.97) (SD 16106.01)	611.46 (US $8.86) (SD 1669.54)	20,507.81 (US $297.21) (SD 23870.69)	43.8
**Education**					
Nil	2002.75 (US $29.02) (SD 4954.36)	2423.38 (US $35.12) (SD 6753.14)	139.44 (US $2.02) (SD 676.44)	4565.56 (US $66.17) (SD 11725.17)	11.3
Up to Primary	2307.03 (US $33.44) (SD 336.98)	3396.02 (US $49.22) (SD 6558.87)	150.78 (US $2.19) (SD 425.99)	5853.83 (US $84.84) (SD 8456.74)	15.6
Up to Secondary	3756.99 (US $54.45) (SD 8041.34)	7665.89 (US $111.10) (SD 15952.47)	429.91 (US $6.23) (SD 1065.17)	11,852.80 (US $171.78) (SD 18410.35)	23.2
Senior Secondary and above	7701.28 (US $111.61) (SD 10578.15)	13,618.72 (US $197.37) (SD 14576.52)	838.03 (US $12.15) (SD 1741.75)	22,158.03 (US $321.13) (SD 21863.13)	40.2
**Birth Parity**					
Less than 2	4631.88 (US $63.22) (SD 8601.45)	8713.79 (US $126.29) (SD 16179.48)	489.39 (US $7.09) (SD 1261.17)	13,835.06 (US $200.51) (SD 20153.67)	24.8
2 to 4	4488.81 (US $65.06) (SD 8335.93)	7581.85 (US $109.88) (SD 12440.55)	477.20 (US $6.92) (SD 1241.39)	12,547.86 (US $181.85) (SD 18094.62)	26.0
More than 4	1387.50 (US $20.11) (SD 2185.84)	3993.21 (US $57.87) (SD 8262.31)	26.79 (US $0.39) (SD 141.74)	5407.50 (US $78.37) (SD 8922.82)	14.3
**Wealth Quintiles**					
First	2201.85 (US $31.91) (SD 6143.55)	2947.68 (US $42.72) (SD 7173.11)	172.62 (US $2.50) (SD 653.51)	5322.14 (US $77.13) (SD 11982.22)	21.4
Second	2084.94 (US $30.22) (SD 2798.56)	6239.29 (US $90.38) (SD 18601.39)	464.88 (US $6.74) (SD 1246.27)	8789.11 (US $127.38) (SD 19425.96)	21.4
Third	2817.06 (US $40.83) (SD 4342.01)	7982.06 (US $115.68) (SD 12479.56)	266.18 (US $3.86) (SD 644.17)	11,065.29 (US $160.37) (SD 14499.28)	22.6
Fourth	5414.05 (US $78.46) (SD 9098.96)	7241.85 (US $104.95) (SD 10424.165)	449.40 (US $6.51) (SD 1054.18)	13,105.30 (US $189.93) (SD 15332.08)	24.1
Fifth	9250.00 (US $134.06) (SD 12535.64)	14,604.46 (US $211.66) (SD 15286.30)	914.46 (US $13.25) (SD 1901.21)	24,768.92 (US $358.97) (SD 23650.95)	34.1
**Average**	**4338.26 (US $62.87) (SD 8208.20)**	**7787.30 (US $112.86) (SD 13844.17)**	**451.96 (US $6.55) (SD 1211.63)**	**12,577.52 (US $182.28) (SD 18567.94)**	**24.8**

*US $1 was approximately Rs. 69 in 2018

Source: Primary Data

On an average, rural women in Punjab have to spend Rs. 4338.26 (US $62.87) for availing the antenatal services, Rs. 7787.30 (US $112.86) on delivery services and Rs. 421.96 (US $6.55) on postnatal services A rising trend in expenditure on all the three components (individually as well as collectively) is visible with increase in age, education and wealth quintile (Table 2). Compared to expenditure on ANC and delivery, respondents have spent less on PNC primarily because not all the respondents have availed the required post natal services. Among those who have availed postnatal care, a larger proportion reported visiting health facilities to get their child vaccinated.

The indirect cost of treatment ranging between Rs. 200 (US $2.90) and Rs. 2700 (US $39.13) is borne by 8.3% of the respondents and majority of these are from poorer wealth quintiles. In the present study it was found that 72.9% of the respondents belonged to joint family structure and thus, the non earning member female member of the family who performed the duty as a caregiver. The mean overall out-of-pocket expenditure (direct and indirect) for the respondents sums to be Rs. 12,661.93 (US $183.51). Only 3.3% (=14) of the total respondents did not avail any expenditure on maternity in their last pregnancy.

Wide variations in the amount of expenditure by place of health facility are observed in the study and the same are presented in Table 3.

**Table 3:** Classification of Expenditure (Direct cost of treatment) according to type of Health Facility

Type of health facility	Expenditure (Rs.)
**Antenatal Care**	
Government	Rs. 2053.63 (US $29.76) (SD 3891.87)
Private	Rs. 14,511.11 (US $210.31) (SD 12891.18)
Both	Rs. 10,296.77 (US $149.23) (SD 12767.79)
**Delivery**	
Government	Rs. 1437.69 (US $20.84) (SD 9303.72)
Private	Rs. 20,840.44 (US $302.04) (SD 12895.26)
Home	Rs. 3596.15 (US $52.12) (SD 3646.03)
**Postnatal Care**	
Government	Rs. 318.90 (US $4.62) (SD 692.93)
Private	Rs. 1137.50 (US $16.49) (SD 2021.23)

Source: Primary Data

Seeking antenatal care from government facilities results in mean expenditure of Rs. 2053.63 (US $29.76). The cost rises to more than seven times (Rs. 14,511.11 or US $210.31) in private hospitals and to five times (Rs. 10,296.77 or US $149.23) when both government and private facilities are used. The expenditure on delivery is maximum in private hospitals (Rs. 20,840.44 or US $302.04), followed by home deliveries (Rs. 3596.15 or US $52.12) and government deliveries (Rs. 1437.69 or US $20.84). The expenditure on PNC in private facilities is more than three times the expenditure on PNC in public facilities.

Respondents were probed for the reasons which forced them to utilize private health services. Among many reasons, one prominent factor governing the choice has been the perceived difference in the quality of services offered at private and public facilities where it is believed that private providers offer services of higher quality than the services offered by public providers. Even the women who have availed services from government institutions in this study complained of apathetic behavior of hospital staff and poor infrastructure. In certain cases a single occupancy hospital bed had to be shared by two patients. There prevails a general mistrust on the efficiency of government facilities as women with complications have preferred to get themselves treated in private facilities. Some respondents were forced to seek delivery services at private facilities either because government facilities failed to treat them due to infrastructural bottlenecks or they were referred to far off facilities. Poor past experience of government hospitals has also compelled women to opt for private deliveries. It is observed in the study that respondents with history of miscarriage or still births while utilizing government health facilities have used private health facilities for their current delivery. There were 149 households who majorly relied on non-agricultural wage labor for their source of income while only 54 households had regular salaried job as their main source of income. In the absence of definite employment, using private health facilities put additional burden on the pocket.

### Incidence of catastrophic expenditure

Expenditure on maternity services is taken to be catastrophic if it is 10% or more of the yearly income of the household. Among the total respondents, ANC, delivery and PNC services necessitated catastrophic spending for one-fourth of the respondents. A detailed classification of extent of catastrophic expenditure by background characteristics of respondents is presented in Table 2. The percentage of women bearing catastrophic maternity expenditure increases with increasing educational attainment, advances in maternal age and increase in wealth quintile. To understand the determinants of catastrophic expenditure, logistic regression has been made use of and the results are presented in Table 4.

**Table 4:** Results of Logistic Regression

Variables	Uni-variable Analysis				Multivariable Analysis			
	Unadjusted Odds Ratio	***P*** value	95% C.I		Adjusted Odds Ratio	***P*** value	95% C.I	
			Lower	Upper			Lower	Upper
**Intercept**					0.006	0.000		
**District**^**a**^								
Gurdaspur (*n* = 60)	1.084	0.841	0.493	2.383	0.622	0.490	0.161	2.398
Hoshiarpur (*n* = 60)	1.084	0.841	0.493	2.383	0.389	0.193	0.0938	1.613
Jalandhar (*N* = 60)	1.572	0.247	0.731	3.381	0.810	0.755	0.216	3.045
Ludhiana (*n* = 60)	0.389	0.047	0.153	0.989	0.467	0.284	0.116	1.882
Patiala (*n* = 60)	0.506	0.130	0.210	1.221	0.502	0.324	0.127	1.978
Tarn Taran (*n* = 60)	0.506	0.130	0.210	1.221	0.556	0.364	0.157	1.973
**Religion**^**b**^								
Sikhism (*n* = 278)	1.002	0.994	0.608	1.652	0.657	0.395	0.249	1.731
Other# (*n* = 25)	0.958	0.934	0.349	2.629	0.087	0.063	0.007	1.138
**Social Group**^**c**^								
General (*n* = 101)	3.333	0.000	1.997	5.563	3.006	0.019	1.202	7.517
Other Backward Class (*n* = 55)	1.449	0.300	0.718	2.924	0.900	0.863	0.274	2.959
Do not know (*n* = 14)	2.601	0.100	0.831	8.138	2.109	0.549	0.183	24.287
**Wealth Quintiles**^**d**^								
First (*n* = 84)	0.365	0.010	0.170	0.787	22.570	0.000	4.632	109.968
Second (*n* = 84)	0.598	0.148	0.298	1.201	18.780	0.000	4.571	77.164
Third (*n* = 84)	0.913	0.787	0.473	1.764	17.027	0.000	4.520	64.144
Fourth (*n* = 84)	0.827	0.576	0.424	1.611	7.593	0.001	2.172	26.544
**Age group**^**e**^**(years)**								
20–30 (*n* = 354)	1.460	0.558	0.412	5.169	0.715	0.694	0.135	3.790
30 and above(*n* = 48)	3.889	0.051	0.994	15.220	0.845	0.866	0.118	6.022
**Education**^**f**^								
Up to Primary (*n* = 64)	1.458	0.459	0.537	3.957	0.723	0.647	0.181	2.895
Up to Secondary (*n* = 168)	2.381	0.038	1.050	5.396	1.168	0.809	0.331	4.123
Senior Secondary and above (*n* = 117)	2.287	0.000	2.321	12.045	1.591	0.522	0.384	6.588
**Birth Parity**^**g**^								
2 to 4 (*n* = 227)	1.062	0.798	0.670	1.684	1.743	0.158	0.805	3.775
More than 4 (*n* = 115)	0.504	0.229	1.65	1.538	0.723	0.722	0.121	4.307
**ANC Place**^**h**^								
Private Health Facility (*n* = 98)	12.337	0.000	7.281	20.904	5.767	0.000	2.405	13.832
**Delivery Place**^**i**^								
Private Health Facility (*n* = 132)	24.902	0.000	13.648	45.435	8.542	0.000	3.108	23.477
Home (*n* = 17)	2.717	0.216	0.557	13.250	2.989	0.222	0.515	17.340
**PNC Place**^**j**^								
Government Health Facility (*n* = 127)	0.705	0.389	0.318	1.562	0.850	0.750	0.313	2.310
Private Health Facility (*n* = 108)	17.956	0.000	9.692	33.267	5.390	0.001	2.019	14.387

^a^Amritsar (*n* = 60) = Reference Group ^b^ Hinduism (*n* = 117) = Reference Group ^c^ Scheduled Caste (*n* = 250) = Reference Group ^d^Fifth (*n* = 84) = Reference Group ^e^ Less than 20 (*n* = 18) = Reference Group ^f^Nil (*n* = 71) = Reference Group ^g^Less than 2 (*n* = 115) = Reference Category ^h^Government Health Facility (*n* = 322) = Reference Group ^i^Government Health Facility (*n* = 271) = Reference Group ^j^None (*n* = 185) = Reference Group (It includes those women who did not have any checkup post discharge from facility and those who delivered at home) #Islam and Christianity

Source: Primary Data

Table 4 presents the results of both uni-variable analysis (unadjusted odds ratio) and multivariable analysis (adjusted odds ratio). In this section, the results of multivariable analysis are discussed. After controlling for other variables in the model, no significant association is seen for the probability of incurring catastrophic maternity expenditure with district, religion, maternal age, education or birth parity. Women belonging to general category^1^ are more likely than their counterparts belonging to Scheduled Caste (SC) to spend catastrophically. Compared to the richest quintile, the odds are higher for other quintiles to bear catastrophic maternity expenditure, with the odds being highest for the poorest quintile and declining gradually. Women who utilized private facilities (partially or solely) for availing antenatal care are more likely to spend 10% or more of their family income compared to women who utilized government facilities alone. Compared to women who delivered at government hospitals, the odds are higher for women delivering at private hospitals. Women who availed post natal care at private facilities are more likely to incur catastrophic expenditure compared to women who received none.

**Fig. 1:**
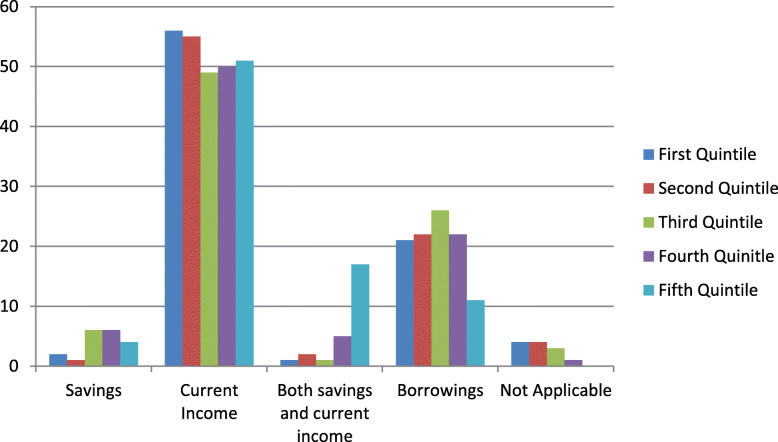
Coping mechanisms for meeting OOPE

Households use various strategies to cope with the expenditure resulting in case of health emergency. These strategies can be utilizing current income or past savings; borrowing from friends or relatives or from money lender; or borrowing from bank using collateral [19]. The coping mechanisms used by the respondents are presented in Fig. 1.

It can be observed from the figure that respondents irrespective of their quintile class have majorly relied on their current income to finance the maternity expenditure. The second most used coping strategy amongst the respondents has been dependence on borrowings from friends/relatives or others. It can be noticed from the figure that usage of borrowed funds is more amongst the respondents belonging to lower wealth quintiles indicating that the need to borrow declines for respondents belonging to higher wealth quintiles given their wider income means. A further look at Fig. 1 reveals that one-fourth of the respondents belonging to the most poor wealth quintile have resorted to borrowing, the percentage increases to 26.2% for the second quintile and 31% for the third quintile. The percentage then declines to 26.2% for fourth quintile and to 13.2% for the least poor quintile. Past savings have been used in case of 19 respondents and there were 26 respondents who relied on both current income and past savings to finance the health expenditure. The usage of savings or both income and savings is comparatively more amongst higher wealth quintiles than the lower wealth quintiles. Of the 102 respondents who have resorted to borrowings, 60 respondents have reported borrowing from their friends or relatives. Borrowing from money lender or bank is reported in case of 37 and one respondent respectively. In case of remaining four respondents, the respondents’ husbands have borrowed from their respective employers. The category not applicable relates to respondents with no expenditure on maternity.

### Entitlements under Janani Shishu Suraksha Karyakaram

**Fig. 2:**
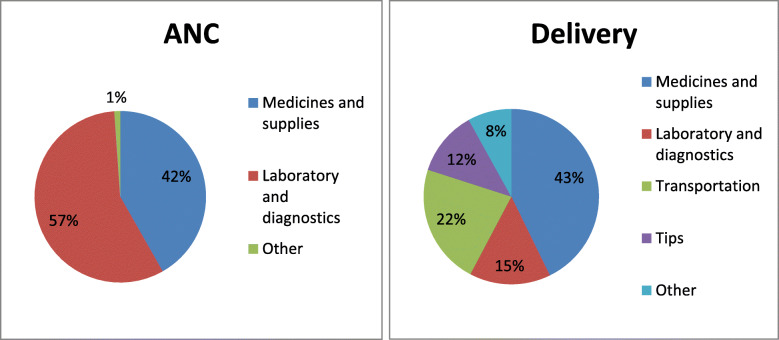
Classification of Expenditure according to Components of Care

The scheme Janani Shishu Suraksha Yojana entitles beneficiaries to free maternity services including ANC, delivery and PNC at government facilities. In this section an analysis is made regarding the efficacy of the scheme in curtailing out of pocket expenditure incurred by the pregnant women. Among 420 respondents of the study, 322 respondents have availed ANC services solely from government hospitals and of these 322 respondents, 84.2% (*n* = 271) are found to bear expenditure on one or the other component. Although the scheme has a provision of free diagnostics and free drugs and consumables, 78.8% respondents are found to bear the diagnostics’ costs and more than one-fourth respondents have been reported to be spending on getting the prescribed medicines. Figure 2 presents classification of total expenditure on ANC and delivery according to components of care.

Of the total expenditure borne on ante natal services by 271 respondents, the spending on laboratory and diagnostics constitutes the largest proportion (57%). Spending on laboratory and diagnostics is followed by drugs and consumables which accounts for 42% of the total expenditure while spending on other (consultation, transport and file charges) constitute a menial 1 % of the total expenditure.

Among the 271 government deliveries, in case of 68.6% deliveries (*n* = 186) out of pocket expenditure has been borne by the beneficiaries. Under JSSK, beneficiaries are provided free transport facility from home to government health institutions and drop back after delivery. Further if the beneficiary is to be referred to other health facility, then free transport facilities are made available. With only 25 respondents being provided with two way transport facilities, the remaining 246 were left to arrange transportation on their own. While 133 respondents are noted to incur expenditure on transport, others have made use of their own/relative’s vehicles. Of the 51 beneficiaries referred to other facilities, 38 were provided with free transport facilities. Informal payments in the form of tips to the hospital staff have been borne by 70 respondents (25.8%) and 52 respondents have reported to spend on medical bills. Of the spending on delivery borne by 186 respondents, expenditure on medicines and supplies contributes the most to the total expenditure (43%) followed by expenditure on arranging transportation (22%) (Fig. 2). Bills on laboratory and diagnostics services constitute 15% of the total expenditure and tips paid to the hospital staff account for 12%. Payments made in the form of charges of consultation, food, tests and room rent constitute 8% of the expenditure. Regarding expenditure on PNC, spending mainly constitutes medicines purchased at the time of discharge from the facility or the post discharge checkups. Such expenditure has been incurred by 52 respondents in the range of Rs. 100 and Rs. 5000.

## Discussion

The present study examines the burden of maternity expenditure in rural areas of Punjab (one of the prosperous states of India) where out of 420 respondents, 96.7% have to bear expenditure irrespective of the type of facility, raising serious questions about the government efforts to curtail OOPE. The spending increases with increase in age at the time of conceiving and educational qualification. The indirect cost component was borne by a relatively small percentage of respondents. While the government facilities are more commonly used for pre-partum services, the usage of private facilities for delivery services increases with age, education and wealth quintile. Wide disparities in the expenditure incurred in the two types of facilities exist where cost of availing the three components of maternity care, namely, ANC, delivery and PNC is seven times, 14 times and three times more respectively in private facilities when compared with public facilities. Household income rather than consumption expenditure has been used to compute catastrophic health expenditure primarily because of the inability of respondents to provide reliable data on consumption expenditure in the absence of definite source of employment, since majority of the respondents relied on non-agricultural wage labor for their income means. One significant determinant of catastrophic maternity expenditure emerged in the study is the type of facility used and as observed from the results of logistic regression, the odds of incurring catastrophic expenditure increase with the utilization of private facilities and thus it becomes relevant to examine the factors driving the choice of health institution. Among many reasons, one prominent factor governing the choice has been the perceived difference in the quality of services offered at private and public facilities where it is believed that private providers offer services of higher quality than the services offered by public providers. Even the women who availed services from government institutions in this study have complained of apathetic behavior of hospital staff and poor infrastructure where in certain cases a single occupancy hospital bed had to be shared by two patients. Incivility and indifference of public provider staff is a major factor pushing away health care seekers especially those coming from the poor and marginalized sections [20]. There prevails a general mistrust on the efficiency of government facilities as women with complications preferred to get themselves treated in private facilities. Some respondents were forced to seek delivery services at private facilities either because government facilities failed to treat them due to infrastructural bottlenecks or they were referred to far off facilities. Poor past experience of government hospitals also compelled women to opt for private deliveries. Women tend to shift to private facilities after their first treatment in public care [21]. Another phenomenon currently prevailing is the increasing share of caesarean section births in the total deliveries, especially in private facilities [22–24]. Caesarean section rates higher than 10% at population level imply utilization of procedure for reasons other than life saving [25]. In over-loaded and weak health systems, unnecessary caesarean sections are known to pull resources away from other services. In addition, unnecessary caesarean sections can also lead to adverse maternal and infant health outcomes. With nearly 60% of deliveries in private facilities being caesarean in the present study, households are additionally burdened. As observed from the study, it is the respondents belonging to lower wealth quintiles who have majorly used their current income and borrowing for meeting the childbearing expenditure, putting them into a greater risk of being reduced to a state of penury. Savings for untoward times as observed is a characteristic of rich, not of those struggling to meet their both ends. Although Punjab ranks amongst the richest states in India, it has no policy of its own aimed at improving the maternal health outcomes. A conditional cash incentive programme was initiated in the state way back in 2011, but it was eventually pulled out due to cash crunch. Fourth round of India’s National Family Health Survey in [26] puts the average OOPE per delivery in public facility in rural Punjab at Rs.2043 (US $29.61); it doesn’t provide information on expenditure incurred on ANC and PNC. The present study reports expenditure incurred on all three components (ANC, delivery and post delivery checkups) in government facilities to be Rs. 3810.22 (US $55.22). Though the Government of India made a concerted effort in the direction of improving maternal health outcomes by introducing Janani Suraksha Yojana (a conditional cash transfer programme entitling pregnant women to monetary incentive for delivery in government or accredited private facilities) under the umbrella of National Rural Health Mission in 2005, the amount of incentive has been insufficient to cover the cost of care.

JSSK was launched with the vision to eliminate out of pocket expenditure of pregnant women; although the situation has not improved much. Though the spending may not be catastrophic for all women having public institutional births, nevertheless a significant amount of money goes in availing services which are deemed to be free. Though maternal care services are touted as free at public government facilities, many households bear OOPE mainly because of unavailability of medicines and diagnostics facilities [18, 27]. Rather than enduring interminable waits with hordes of other patients for getting ultrasound done during pregnancy at public diagnostic facilities, respondents have preferred to utilize private diagnostic centers involving expenditure. Malpractices of government healthcare providers too have resulted in significant expenditure to certain beneficiaries where in they were asked to get their diagnostics done by the private facility designated by government doctor, in the absence of which treatment would be denied. Insufficient human and physical infrastructure at public facilities compels people to approach private diagnostic facilities where costs are high [28]. Round the clock transport services are not available and even when provided these are restricted to one-way service, either from home to facility or drop back home after delivery.

## Conclusion

Scanty budgetary deployments for existing health facilities, and insufficient investment and consequent underdevelopment of new health facilities have resulted in abysmal performance of the public healthcare sector [29]. In order to generate additional resources for this struggling sector, India must reprioritize its policies both at macro and sector level. Along with addressing the issues of poor accountability, infrastructural constraints and corruption in public facilities, unregulated expansion of private sector needs to be thwarted which continues to attract majority of OOPE. Although when studied in isolation the results of univariable analysis reveal that respondents belonging to lowest wealth quintile are less likely than their counterparts from highest wealth quintile to spend catastrophically, a contrasting picture emerges when the influence of all variables is taken together in multivariable analysis. The results of multivariable analysis reveal that after adjusting for characteristics such as place of delivery, educational status, etc., women at the bottom of socio-economic status are found to have higher probability of incurring catastrophic expenditure. The strategy towards curbing the impoverishment caused by maternity costs will require a proactive role of the states in framing policies making maternity services easily accessible and affordable. In India’s pursuit of achieving the targets under Sustainable Development Goals, states will have to a play critical role since the implementation of social sector programs is squarely in the realm of state governments. An all-inclusive package of services along with improving the institutional capacity in logistics, better management of human resources and reliable accountability mechanisms will provide the way forward.

## Supplementary Information

**Additional file 1.** Interview Schedule.

## Data Availability

The present study is a part of doctoral thesis which is yet to be submitted. The datasets used for the study are available from the corresponding author on reasonable request.

## Abbreviations

JSSK: Janani Shishu Suraksha Karyakaram
OOPE: Out-of-pocket Expenditure
ANC: Antenatal Checkup
PNC: Postnatal Checkup

## Footnotes

1 Refer to castes whose members are on average economically and socially ahead of others.
