# Supplementary material for: Analysing the expenditure on childbearing: a community-based cross-sectional study in rural areas of Punjab (India)
Source: BMC Health Serv Res. 2021 Jan 21;21:76. doi: 10.1186/s12913-021-06075-2 (PMC7818910; doi:10.1186/s12913-021-06075-2)
Supplement: Supplementary file 1 — Additional file 1. Interview Schedule. [file 12913_2021_6075_MOESM1_ESM.docx]

***My name is Niharika Mahajan and I am working as a Research Fellow at Punjab School of Economics, Guru Nanak Dev University, Amritsar. For achieving the objectives of my PhD research work, I need certain information that can be supplied by you. The information provided by you will be kept confidential and will be used strictly for academic purposes only.***

Household’s Information Schedule

1. District: __________
2. Block: ___________
3. Village: __________
4. Name of the head of the household: _________
5. Contact No.: ____________
6. Religion of the head of the household: ___________

Codes:

01: Hinduism 02: Islam 03: Christianity

04: Sikhism 05: Jainism 06: Buddhism

07: No religion 08: Other

1. Caste of the head of the household: ____________

Codes:

01: General 02: Schedule Cast/Schedule Tribe

03: Other Backward Class 04: Do not know/do not want to respond

1. Ownership of house: _________________

Codes:

01: Owned 02: Rented

1. Does your household own any land?_____________

Codes:

01: Yes 02: No

If yes, then how much? __________________________

1. Occupation of the head of the household: _________

Codes:

01: Agricultural wage labourer

02: Non-agricultural wage labourer

03: Self employed/ Regular salaried/ Regular wage employee

04: Rentier, Pensioner, other remittance recipient

05: Domestic chores

06: Do not work

07: Other

1. Does any other member of the household besides the head work? _______

Codes:

01: Yes 02: No

If yes, what is the occupation? __________________

Codes:

01: Agricultural wage labourer

02: Non-agricultural wage labourer

03: Self employed/ Regular salaried/ Regular wage employee

04: Rentier, Pensioner, other remittance recipient

05: Domestic chores

06: Do not work

07: Other

1. What is the monthly income of the household from all sources?

__________________________________________________________

1. Type of dwelling: ___________

Codes:

01: Kutcha 02: Semi Pucca 03: Pucca

1. Number of rooms used for sleeping ________________________
2. Does this household have a separate kitchen, which is used as a kitchen?

01: Yes 02: No

1. Type of fuel used for cooking: ___________

Codes:

01: Electricity 02: Biogas/Gobar Gas 03: LPG/Natural Gas 04: Kerosene 05: Wood 06: Agricultural crop waste

07: Dung cakes 08: Other

1. Source of drinking water*: _________

Codes:

01: Improved 02: Unimproved

*Improved: Piped water into dwelling; Piped water to yard/plot; Public tap or standpipe; Tubewell or borehole; Protected dug well; Protected Spring; Bottled water (provided a secondary source of improved water exists for other uses such as personal hygiene, cooking, etc.); Rainwater

Unimproved: Unprotected spring; Unprotected dug well; Cart with small truck/drum; Tanker truck; Surface water

1. Do you treat the water in any way to make it safe for drinking? ___________

01: Yes 02: No

If yes, then how? ________________________________________________________

1. Kind of toilet facility**: __________

Codes:

01: Improved 02: Unimproved

**Improved: Flush/Pour flush to Piped sewer system, septic tank, pit latrine; Ventilated improved pit latrine; Pit latrine with slab; Composting toilet

Unimproved: Flush/Pour flush to elsewhere, unknown place; Pit latrine without slab/Open slab pit; Bucket; Hanging toilet/hanging latrine; Other; No toilet facility/Uses open space/field

1. Do you share this toilet facility with some other household? ______

Codes:

01: Yes 02: No

1. Assets available in the household:

Tv ____________

Fridge ____________

Air Conditioner ____________

Washing Machine ____________

Motorcycle ____________

Codes:

01: Yes 02: No

1. Household Information Schedule

| S.No. | Residents of the Household (Name) | Relationship with the head of the Household  Codes:  01: Head  02: Spouse  03: Son or Daughter  04: Son-in-law or Daughter-in-law  05: Grandchild  06: Parent  07: Parent-in-law  08: Brother or Sister  09: Brother-in-law or Sister-in-law  10: Niece/Nephew  11: Other | Sex  Codes:  01: Male  02: Female | Age | Marital Status  Codes:  01: Currently Married  02: Widowed  03: Divorced/Separated  04: Never Married | Education#  Codes:  01: Nil  02: Up to primary  03: Up to secondary  04: Senior Secondary and above |
| --- | --- | --- | --- | --- | --- | --- |

Interview Schedule for Ever-married women aged 15-49 years who delivered in the one year preceding the date of survey

1. Name: __________________________________
2. Date of last delivery:____________________
3. Do you work somewhere? ____________

Codes:

01: Yes 02: No

If yes, where are you working? ___________________________________

1. Were you working before pregnancy? _______________

Codes:

01: Yes 02: No

1. Do you own any of the following?

Property (alone or jointly with others) __________

Bank Account __________

Mobile Phone __________

Codes:

01: Yes 02: No

1. Do you use the following for yourself?

Bank Account ________

Mobile Phone ________

Codes:

01: Yes 02: No 03: Not Applicable

1. Age at the time of marriage:________________
2. How many children you have? ___________________
3. Age at the time of first pregnancy? __________________
4. Age at the time of last pregnancy? ________________
5. Are you aware of the following scheme:

Janani Shishu Suraksha Karyakaram ________

Codes:

01: Yes 02: No

1. Out of the following entitlements for pregnant women under JSSK, about which you are aware of?

Free Delivery _________

Free Caesarean section _________

Free drugs and consumables _________

Free diagnostics (Blood, Urine tests and USG) _________

Free Diet during stay in health institution (up to 3 days for normal delivery and 7 days for C-Section) _________

Free provision of blood _________

Free transport from home to health institution, between health institutions in case of referrals and drop back home _________ Exemption from all kinds of user charges (Also covers antenatal and postnatal period complications) _________

Codes:

01: Yes 02: No

1. Was your last pregnancy registered? ________

Codes:

01: Yes 02: No

1. With whom did you register your pregnancy? ________

Codes:

01: Government Doctor 02: ANM 03: Accredited Social Health Activist (ASHA) 04: Private Doctor 05: Anganwadi Worker 06: Other

1. Did you get a card (Mother & Child Protection/any other similar card) at the time of registration? _________

Codes:

01: Yes 02: No

1. How many months were you pregnant when you first registered your pregnancy? ______________
2. Where did you receive Antenatal care for last pregnancy? _________

Codes:

01: Government Health Facility (SHC, PHC, CHC, Rural Hospital, Urban Health Centre/Urban Health Post/Urban Family Welfare Centre, Government Hospital or Dispensary, Anganwadi Centre/ICDS Centre, Ayush Hospital, NGO/Trust Hospital/Clinic)

02: Accredited Private Hospital

03: Private Health Facility (Private Hospital/Clinic)

04: Other (own home, parents’ home, other home, other)

1. Number of times antenatal checkups received during pregnancy associated with this birth? ____________
2. Did you receive Tetanus Toxoid injection during pregnancy associated with this birth? _______

Codes:

01: Yes 02: No

If yes, then how many? ______

1. For how many days and how much Iron Folic Acid tablets/bottles did you consume during last pregnancy?

Number of tablets_________ Number of days__________

Number of bottles_________ Number of days__________

1. From where did you get Iron Folic Acid tablets/syrup? ____________________________
2. Why did you not go for any ANC? _____________

01: Not necessary 02: Too far/no transport 03: Not customary

04: Poor Quality Service 05: Cost too much 06: Family did not allow

07: Lack of knowledge 08: No time to go 09: Not Applicable

10: Other

1. Number of visits per week made by the ASHA during pregnancy? ___________________
2. Did ASHA advice you about following?

Birth preparedness ________________

Safe delivery __________________

Feeding practices __________________

Immunization __________________

Family planning __________________

Codes:

01: Yes 02: No

1. Where did your last delivery take place? __________

Codes:

01: Government Health Facility 02: Accredited Private Hospital

03: Private Health Facility 04: Other (own home, parents’ home, other home, other)

1. Who decided about the place of delivery? _____________________________________
2. What were the reasons for not going to health facility for delivery? __________

Codes:

01: Not necessary 02: Too far/no transport 03: Not customary

04: Poor Quality Service 05: Cost too much 06: Family did not allow

07: Lack of knowledge 08: No time to go 09: Not Applicable

10: Other

1. Who motivated you to go for institutional delivery? ____________

Codes:

01: Doctor 02: ANM 03: ASHA

04: Anganwadi Worker 05: Other health personnel 06: Husband 07: Relatives/Friends 08: Self 09: Other

1. Were you issued referral slip by ASHA/ANM/MO in case of delivery at accredited private hospital? _____________

Codes:

01: Yes 02: No 03: Not Applicable

1. What was the reason for opting for private hospital for delivery? _____________________________________________________________________________
2. Did you face any difficulty while using public health facilities?

_____________________________________________________________________________

1. Was the delivery normal or caesarean ? __________________

Codes:

01: Normal 02: Caesarean

1. Who conducted your last delivery? ___________

Codes:

01: Doctor 02: ANM/Nurse/Midwife/LHV 03: Dai 04: Friends 05: Other health personnel 06: Other

1. How long did you stay in the hospital after delivery? _____________________________________
2. Did ASHA stayed with you at the health facility till you were discharged?

Codes:

01: Yes 02: No 03: Not applicable

1. Was diet provided to the patient in the hospital?

Codes:

01: Yes 02: No 03: Not applicable

1. Was diet provided to the patient throughout the stay in the hospital?

Codes:

01: Yes 02: No 03: Not applicable

1. Who arranged the transportation to take you to the health facility for delivery? ____________

Codes:

01: Doctor 02: Anganwadi Worker 03: ANM 04: ASHA

05: Husband 06: Relatives/Friends 07: Self 08: NGO

09: Other

1. Who arranged transportation for drop back from institution to home after delivery? ________

Codes:

01: Doctor 02: Anganwadi Worker 03: ANM 04: ASHA

05: Husband 06: Relatives/Friends 07: Self 08: NGO

09: Other

1. From the time you registered your pregnancy till the time of delivery, were you referred to some other hospital? ______________

Codes:

01: Yes 02: No

If yes, what was the reason given? ______________________________________________

Who arranged transportation to and back from referred hospital? ____________

Codes:

01: Doctor 02: Anganwadi Worker 03: ANM 04: ASHA

05: Husband 06: Relatives/Friends 07: Self 08: NGO

09: Other

1. Did you have any check up within 24 hours after delivery? _______________

Codes:

01: Yes 02: No

1. How many days after the discharge from hospital first checkup took place? ________________
2. Where did the first check up take place? ____________

Codes:

01: Government Health Facility 02: Accredited Private Hospital

03: Private Health Facility 04: Other (own home, parents’ home, other home, other)

1. How many total post natal check-ups you received? _____________
2. Why did you not avail any PNC? __________

01: Not necessary 02: Too far/no transport 03: Not customary

04: Poor Quality Service 05: Cost too much 06: Family did not allow

07: Lack of knowledge 08: No time to go 09: Not Applicable

10: Other

1. In the two months post delivery, did any health personnel, anganwadi worker or a traditional birth attendant paid you a visit? _________________

Codes:

01: Yes 02: No

1. Can you provide me details on the amount paid on the following, if any

Direct Medical Expenditure

Room Rent________________________________

Consultation Charges________________________

Medicines & Supplies (prescribed from the facility)____________________

Laboratory & Diagnostic Services______________________________

Blood Transfusion (including expenditure for arranging blood)_______________________

Newborn Care___________________

Direct Non-medical Expenditure

Transportation (hiring vehicle to and from the facility)_____________________

Expenses on food during stay in facility___________________

Tips for getting services____________________

Other (including all expenditure that the women could not classify under the specific categories)______________________________________________________

Indirect Costs

Wages lost during hospital stay by both the patient and patient accompaniers_____________________________

1. Cost incurred on antenatal checkups __________________________________
2. Cost incurred on delivery ______________________________________________
3. Cost incurred on postnatal checkups ______________________________________
4. For those covered under health insurance, what was the amount refunded? __________________________________________________________
5. How did you meet the expenditure incurred on maternal health services?__________________

01: Savings 02: Wages and Salary 03: Borrowings 04: Sale of assets

05: Other

1. If the expenditure was financed through borrowings, then what was source of borrowings? __________

Codes:

01: Relatives/Friends 02: Money lender 03: Bank 04: Other

1. Any other remarks

______________________________________________________________________________

____________________________________________________________________________________________________________________________________________________________
